# Abnormal Nutritive Sucking as an Indicator of Neonatal Brain Injury

**DOI:** 10.3389/fped.2020.599633

**Published:** 2021-01-12

**Authors:** Sabrina Shandley, Gilson Capilouto, Eleonora Tamilia, David M. Riley, Yvette R. Johnson, Christos Papadelis

**Affiliations:** ^1^Jane and John Justin Neurosciences Center, Cook Children's Health Care System, Fort Worth, TX, United States; ^2^Department of Communication Sciences and Disorders, University of Kentucky, Lexington, KY, United States; ^3^NFANT Labs, LLC, Marietta, GA, United States; ^4^Division of Newborn Medicine, Department of Pediatrics, Boston Children's Hospital, Harvard Medical School, Boston, MA, United States; ^5^Neonatal Intensive Care Unit, Cook Children's Health Care System, Fort Worth, TX, United States; ^6^School of Medicine, Texas Christian University and University of North Texas Health Science Center, Fort Worth, TX, United States; ^7^Neonatal Intensive Care Unit Early Support and Transition (NEST), Developmental Follow-Up Center, Neonatology Department, Cook Children's Health Care System, Fort Worth, TX, United States; ^8^Department of Bioengineering, University of Texas at Arlington, Arlington, TX, United States

**Keywords:** sucking, brain injury, neuroimaging, nutritive, non-nutritive

## Abstract

A term neonate is born with the ability to suck; this neuronal network is already formed and functional by 28 weeks gestational age and continues to evolve into adulthood. Because of the necessity of acquiring nutrition, the complexity of the neuronal network needed to suck, and neuroplasticity in infancy, the skill of sucking has the unique ability to give insight into areas of the brain that may be damaged either during or before birth. Interpretation of the behaviors during sucking shows promise in guiding therapies and how to potentially repair the damage early in life, when neuroplasticity is high. Sucking requires coordinated suck-swallow-breathe actions and is classified into two basic types, nutritive and non-nutritive. Each type of suck has particular characteristics that can be measured and used to learn about the infant's neuronal circuitry. Basic sucking and swallowing are present in embryos and further develop to incorporate breathing *ex utero*. Due to the rhythmic nature of the suck-swallow-breathe process, these motor functions are controlled by central pattern generators. The coordination of swallowing, breathing, and sucking is an enormously complex sensorimotor process. Because of this complexity, brain injury before birth can have an effect on these sucking patterns. Clinical assessments allow evaluators to score the oral-motor pattern, however, they remain ultimately subjective. Thus, clinicians are in need of objective measures to identify the specific area of deficit in the sucking pattern of each infant to tailor therapies to their specific needs. Therapeutic approaches involve pacifiers, cheek/chin support, tactile, oral kinesthetic, auditory, vestibular, and/or visual sensorimotor inputs. These therapies are performed to train the infant to suck appropriately using these subjective assessments along with the experience of the therapist (usually a speech therapist), but newer, more objective measures are coming along. Recent studies have correlated pathological sucking patterns with neuroimaging data to get a map of the affected brain regions to better inform therapies. The purpose of this review is to provide a broad scope synopsis of the research field of infant nutritive and non-nutritive feeding, their underlying neurophysiology, and relationship of abnormal activity with brain injury in preterm and term infants.

## Historical Background

As early as the 1940's researchers began examining the sucking behavior of infants. Before this time, the milk ejection, or “let-down,” reflex was regarded as the predominant method of breast milk transfer (1, 2). Sucking patterns of infants were differentiated based on the frequency and intensity of the sucking, which was found to correlate with whether or not fluid (breastmilk or formula) is present. These patterns became known as *nutritive* and *non-nutritive* sucking (1, 3–5). In the 1950's researchers started to deconstruct the infant suck into two different skills, *suction* and *expression/compression* (6–8). Based on this deconstruction, research in the 1960's focused on evaluating how an infant would modify these two skills to obtain nutrients using lab-made apparatus' that would control when nutrient was released based on the amount of suction or expression (6). These experiments demonstrated the incredible learning ability of an infant's brain to adapt to changing conditions in line with more recent ideas of brain neuroplasticity during the early years of life (9–11). Nurses also began noticing the relationship between feeding as an infant and speaking ability later in life (12). Concurrently, other groups began looking into the relationship between brain injury and sucking; they observed differences between non-nutritive sucking of normal, term infants, and those who experienced perinatal stress with or without neurological signs (3).

The 1970's brought the confirmation that the anatomy and physiology of the infant, feeding on a pure liquid diet, is profoundly different than the adult (13). This paved the way for the field of dysphagia in infants to be studied and treated differently than in adults. The following decade, dysphagia in infants and children became a focus of researchers and clinicians alike as medical care improved outcomes for preterm infants. Also, neuroscientists and speech therapists began to investigate the correlation between sucking pattern in infancy and fine motor skills around 6 months, speech-language delays at 18 months, and developmental delay at 24 months (14). In the 1990's the field broadened significantly to include molecular, developmental, and genetic biology (15, 16).

The new millennia began with trials that confirmed developmental enhancement interventions and physical therapy performed on infants with brain injury were not working well (11, 17, 18), even though success had been seen with older children (19–21). Another compounding factor was identification of infants with brain injury by MRI, which is unreliable as a sole predictor of clinical impairment or prognosis (22–24). Clearly, interventions need to be tailored to the term and preterm infants with brain injury and the injury itself needs other modalities for identification (25). During this time, the idea of neuroplasticity became the topic of numerous studies in cerebral palsy research which clearly demonstrates re-organization of the brain as a result of a prenatal or perinatal brain insult (26–31). Additionally, brain injury repair is being elucidated in both human and animal studies (32–36). These advancements have led to the current research field of identifying brain injury through evaluation of sucking as well as habilitation of sucking in infants to potentially repair brain injury.

## Nutritive and Non-Nutritive Sucking

**Figure 1:**
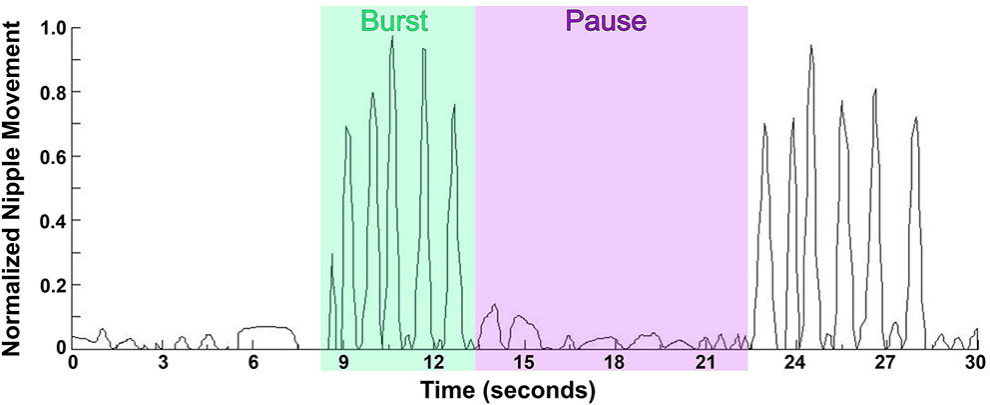
Waveform pattern of NNS. Graph represents the extent of the movement of the lever inside the bottle nipple, measuring the expression component of sucking with 1.0 being the furthest the lever inside the nipple can move, and 0.5 being half the maximum distance. NNS occurs at up to two sucks per second in short, fast bursts lasting anywhere from 2 to 12 s with a pause between bursts of 3–13 s.

Early in the investigations of infant's sucking it became clear that there are two distinct types, namely, *nutritive* and *non-nutritive* sucking. As an infant gains more experience, these sucking patterns mature in strength and efficiency. The variation within an individual is small, however there could be fairly significant interindividual variations. Non-nutritive sucking (NNS) is the primary pattern seen when an infant sucks on a pacifier, his/her thumb, or other objects. NNS occurs at up to two sucks per second (*frequency*) in short, fast bursts (1, 3, 14, 37). The bursts can last anywhere from 2 to 12 s (*burst duration*) with a rest period (pause) between bursts of 3–13 s [(3, 38); Figure 1]. The greatest predictors of a mature NNS pattern is post-menstrual age (PMA) and birth weight (39). As the infant ages, the *frequency* and *burst duration* may increase to a NNS pattern considered more mature, closer to two sucks per second with a duration between 2–8 s and less inter-burst duration and inter-rest period variations, hence a smoother, more regular NNS (38, 39). When breast-feeding, a newborn will begin with NNS until the milk ejection reflex occurs, then will switch to nutritive sucking (40). If NNS is not encouraged, by breast-feeding or pacifier use, for example, it will disappear by 4–5 months of age, however, fascinatingly it can be found in some adults with degenerative cerebral diseases (39).

There are several factors which may affect the different features of a NNS. For example, pacifier characteristics may impact the way an infant will suck on it. The thickness of the silicone of a pacifier, therefore, its stiffness, will affect the NNS pattern of infants. Stiffer pacifiers will elicit fewer sucks per burst, up to half as many, and also the strength, or amplitude, of each suck is decreased. The shape of the pacifier or any texture will also profoundly affect the NNS pattern (41–43).

**Figure 2:**
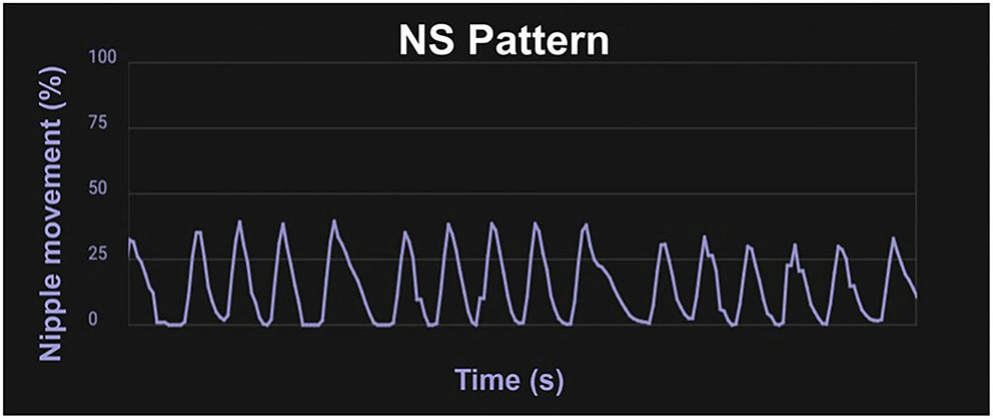
Waveform pattern of NS. Graph from the infant Feeding Solution showing the extent of the movement of the lever inside the bottle nipple measuring the expression component of sucking with 100% being the furthest the lever inside the nipple can move, and 50% being half the maximum distance. A mature NS pattern demonstrates regular, smooth movement about one suck per second.

Nutritive sucking (NS) occurs at a slower pace, about one suck per second (1, 3), and as the feed continues a burst–pause pattern emerges. The first minutes of NS are steady with none or very few short pauses, as the feed continues, bursts appear with a pause between bursts that gets longer toward the end of the feeding [(14, 44); Figure 2]. The rooting reflex, which is the movement of an infant's head toward a touch on their cheek accompanied by mouth gapping, is present at birth for neurologically normal infants born at 32 weeks gestational age (GA) and older, and sometimes even very preterm infants will root before 32 weeks PMA (45). This reflex assists the infant in locating a food source and will disappear around 6 months old (1).

## Development of Normal Sucking Activity

**Figure 3:**
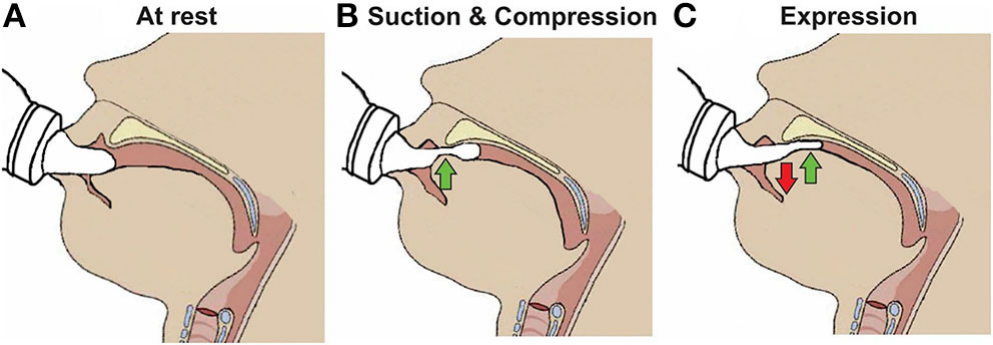
Skills required for sucking in infants. **(A)** Infant at rest with the nipple inside the mouth. **(B)** Suction applied to the nipple to draw further into the mouth to form a teat and the tip of the tongue beginning to compress it. **(C)** Expression of the teat by the tongue movement against the hard palate.

**Figure 4:**
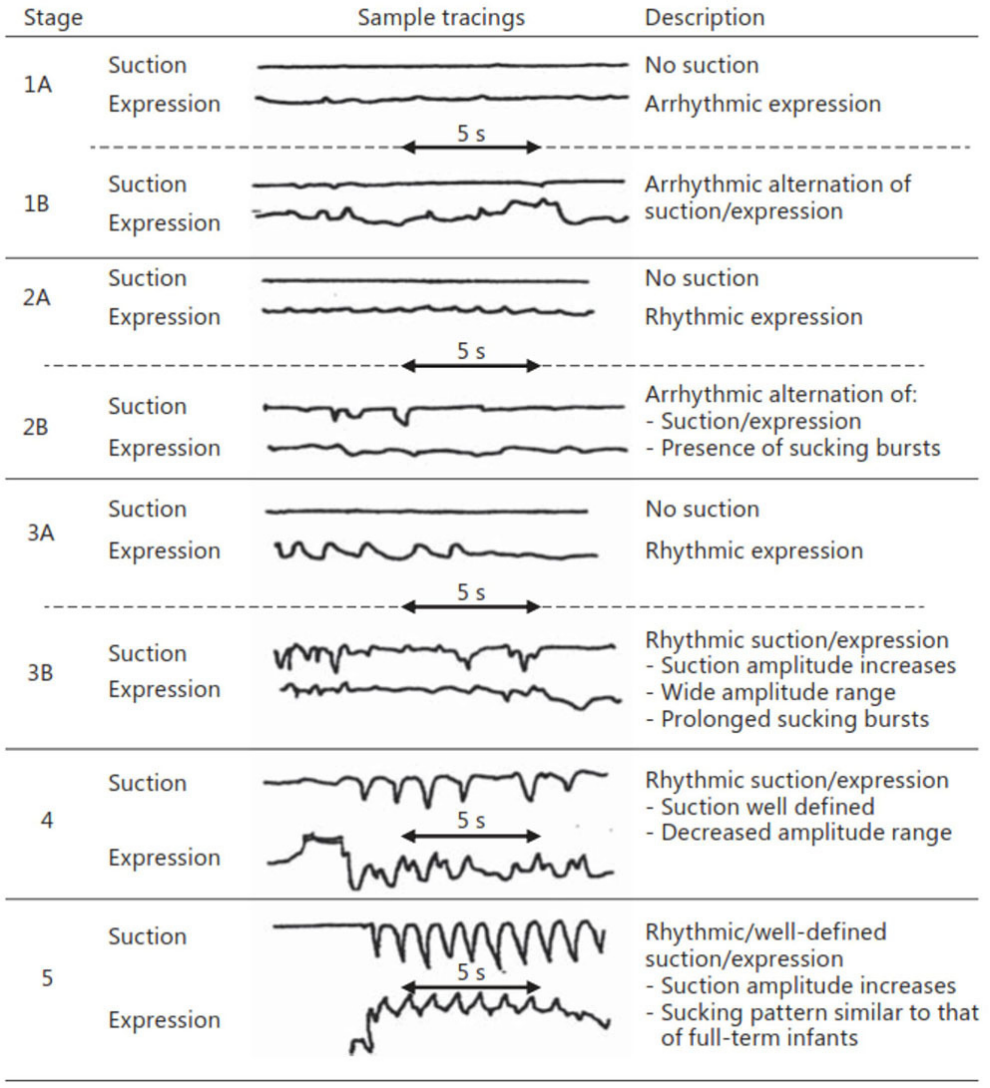
Stages of NS. Early stages (1–3) are seen in preterm infants, while more mature stages (4 and 5) are seen in term infants as well as preterms after enough experience and maturation. Reprinted with permission from Lau (37).

Nutritive sucking is a highly coordinated activity between sucking, swallowing, and respiration (37, 46, 47). Sucking and swallowing skills develop *in utero* as the fetus regulates amniotic fluid levels (14, 16, 48, 49) and must be further developed *ex utero* to incorporate breathing. For both NS and NNS sucking, two skills are required, suction and expression/compression (1, 3, 6, 47, 50). Expression develops first and is the compression or stripping of the tongue against the hard palate to eject liquid (Figure 3). Expression/compression, without the suction component of oral feeding, appears to be present at birth as even very preterm infants as young as 26 weeks GA, have a coordinated 1:1 expression-swallow pattern, albeit slow, and the swallowing process is immature (37). Suction is intraoral negative pressure that draws liquid into the mouth. Suction also requires lowering the jaw to increase the volume of the mouth, closure of the nasal passage by the soft palate, and a tight seal by the lips to prevent air inflow. The development and coordination of these two skills can be measured in five stages: (i) stage 1, no suction and sporadic/arrhythmic expression; (ii) stage 2, no suction or weak, sporadic suction and more organized rhythmic expression pattern; (iii) stage 3, stronger expression, more organized suction/expression pattern emerging; (iv) stage 4, suction is well-defined, suction, and expression strength (amplitudes) becoming more consistent; and (v) stage 5, suction is stronger (increase amplitude), suction/expression has a defined, rhythmic pattern [(47, 51); Figure 4].

## Relationship to Breathing

For healthy adults swallowing is dominant to breathing, which prevents aspiration, and in 75–95% of adults swallowing is initiated during mid-expiration (52, 53). This pause in breathing to swallow is termed swallowing apnea, or deglutition apnea. Infants, however, vary considerably in their swallow-respiration patterns for inter-infant comparisons as well as inter- and intra-feeding comparisons. There are nine possible combinations of swallowing and breathing, with or without pauses: (i) inspiration (I)-swallow-I; (ii) I-swallow-expiration (E); (iii) E-swallow-I; (iv) E-swallow-E; (v) I-swallow-pause (P); (vi) P-swallow-I; (vii) E-swallow-P; (viii) P-swallow-E; and (ix) P-swallow-P. Some studies combine the swallow patterns with pauses into one grouping as a “pause” group (54), others call it “apnea from multiple swallows (AMS).” In addition, some may group patterns by when the swallow occurs, type I having the swallow in-between phases (I-swallow-E and E-swallow-I), type II having the swallow within phases (I-swallow-I or E-swallow-E) and type III being AMS (55–57). As discussed in the next section, the anatomy and physiology of the infant is profoundly different than in the adult and allows feeding to be done simultaneously with breathing, which explains how an infant can have such variable patterns and not aspirate during feeding.

## Anatomy and Physiology

**Figure 5:**
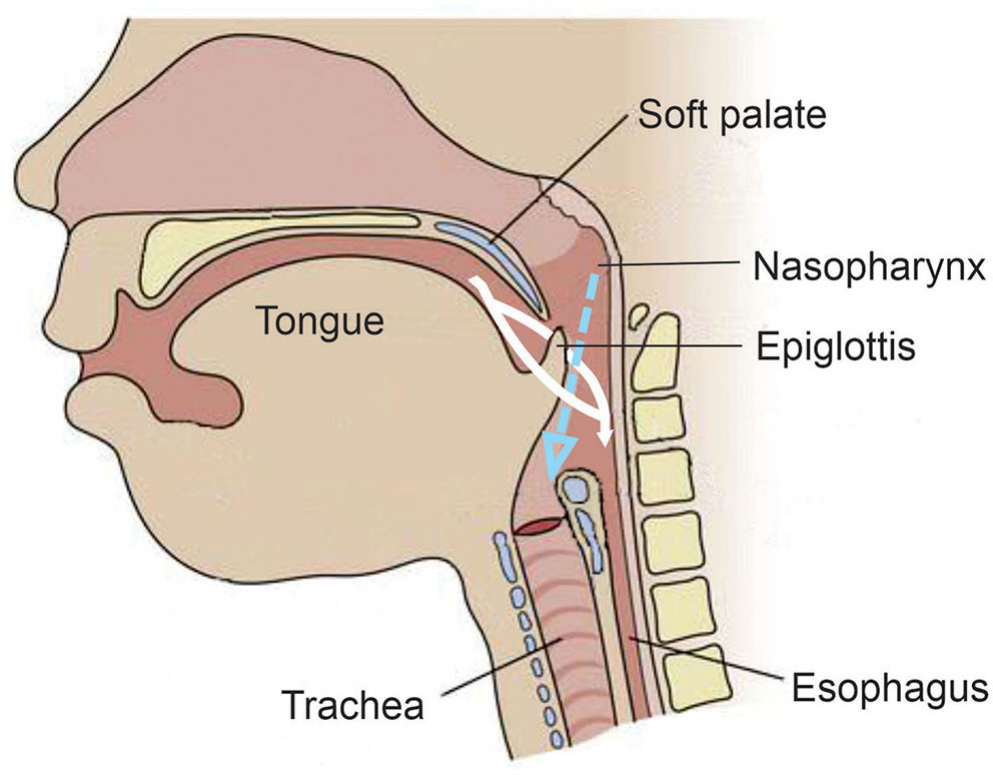
Anatomy and physiology of the infant during feeding. Unlike the adult, the epiglottis moves upward toward the soft palate during feeding. The white indicates the fluid meal and demonstrates how it is made to go around the epiglottis and into the esophagus. The dotted blue arrow indicates the air coming from the nasal passage during the feeding and demonstrates its laminar flow into the trachea.

The evolution from suckle to mastication as the infant matures into childhood develops and changes in anatomy, physiology, and neural networks (15, 16, 48, 52). Unlike adults, an infant's epiglottis moves upward to the soft palate, which allows the trachea to remain open to the nasopharynx to permit constant breathing during sucking. This has been described as the liquid being made to go around each side of the epiglottis and flow into the pharynx and esophagus while still allowing laminar flow of air through the nasopharynx into the trachea [(15, 52, 55, 58); Figure 5]. The brief pause (350–850 ms) in breathing by a sucking infant, deglutition apnea, has been attributed to neuronal control rather than an airway protection mechanism, perhaps in preparation for the more mature swallowing of an adult that requires aspiration prevention (15, 55).

**Figure 6:**
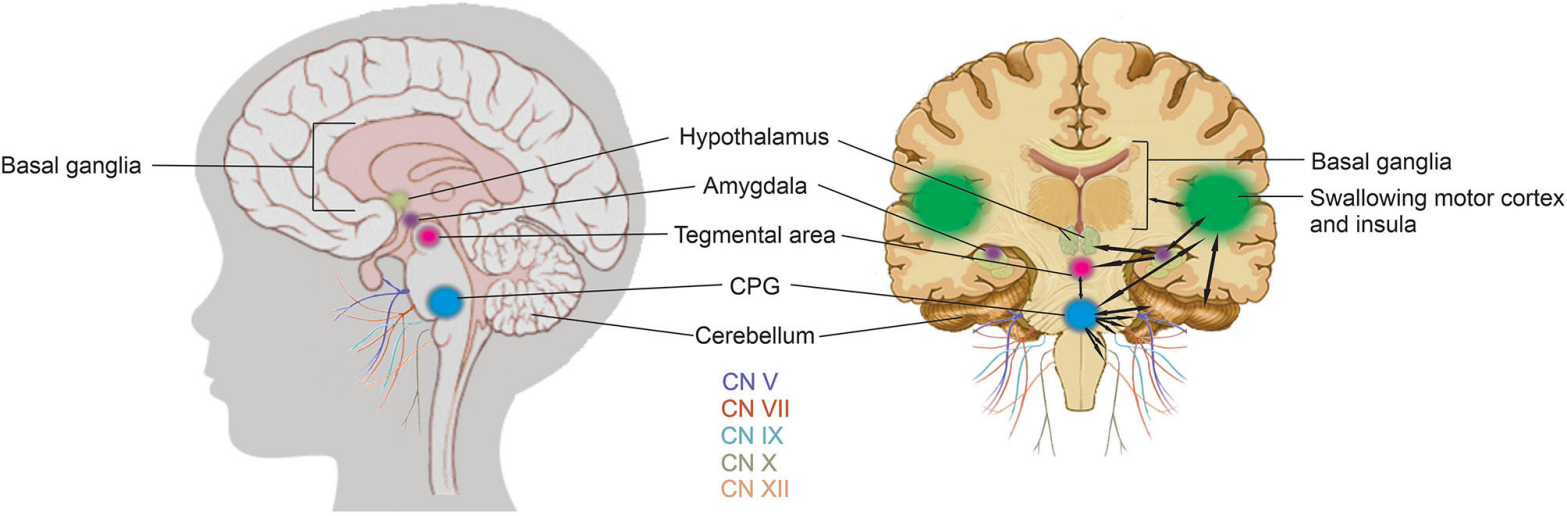
Sucking and swallowing brain network. Sucking and swallowing is a bilateral process in the brain, each hemisphere has been shown to act both contralaterally and ipsilaterally, therefore, for simplicity, the black arrows on the right, coronal view of the brain indicate the complex integration and communication between the areas in only one hemisphere currently believed to be involved in the motor aspects of sucking. CPGs (blue), are at the core of this complex system. A second level of controls includes subcortical structures including the basal ganglia, hypothalamus (pale olive green), cerebellum, amygdala (purple), and tegmental area (pink) of the midbrain with a third level in the suprabulbar cortical swallowing center and insula (green). The cranial nerves (CN) required for the motor process of feeding and swallowing include CN V and VII to move the jaw and facial muscles, CN XII for tongue movement, CN V, IX, and X for movement of the epiglottis, expansion of the uvula and elevation of the hyoid bone and larynx, and CN X for peristaltic movement of the esophageal muscles.

The coordination of swallowing, breathing and sucking is an enormously complex sensorimotor process, requiring five cranial nerves, at least 26 pairs of muscles, the cervical and thoracic spinal cord as well as at least 10 discrete brain areas (48, 52, 53, 58). Due to the rhythmic nature of the suck-swallow-breathe process, these motor functions are controlled by central pattern generators (CPGs) (14, 15, 37, 48, 53, 58). The interneurons of these CPGs are found in the brainstem, specifically the upper medullary and pontine areas, and have been shown to be capable of generating a basic swallow without other input (14, 48, 53). CPGs are at the core of this complex system. A second level of controls includes subcortical structures including the basal ganglia, hypothalamus, cerebellum, amygdala, and tegmental area of the midbrain with a third level in the suprabulbar cortical swallowing center (48). Figure 6 is a graphic representation of the brain network that coordinates sucking and swallowing. For a more in depth review the reader is directed to Hockman et al. (59), Diamant (60), Matsuo and Palmer (52), Mistry and Hamdy (48), Barlow (53), LaMantia et al. (16), Li-Jessen and Ridgway (61), and Maynard et al. (15).

The integration of these three levels of control in the brain are required to coordinate the three phases of NS: (i) oral; (ii) pharyngeal; and (iii) esophageal (15, 47, 48, 58). The oral phase requires cranial nerves (CN) V and VII to move the jaw and facial muscles (to latch onto either breast or bottle nipple) and CN XII for tongue movement to accomplish suction and expression/compression. The pharyngeal phase requires CN V, IX, and X for movement of the epiglottis anteriorly against the soft palate, expansion of the uvula and elevation of the hyoid bone and larynx to accomplish moving of liquid into the pharynx. The esophageal phase requires CN X for peristaltic movement of the esophageal muscles (15, 16, 52, 53, 55). Combined, the sensory and motor neurons that contribute to these five cranial nerves, sensory or motor relay nuclei in the brainstem, and their interconnections constitute the sucking neural circuit (15). This is a highly dynamic and constantly changing circuit based on chemosensory and experiential inputs.

## Sucking Activity In Preterm Infants

Healthy, term infants are, in general, born with the basic skills to perform NS, however, a preterm infant is denied the additional time *in utero* to develop. After gestational age of 28 weeks, it seems that sucking and swallowing are sufficient enough to begin oral feeding; however, they are not coordinated with breathing usually until 32 weeks PMA, with significant improvement around 34 weeks PMA (62, 63). In addition to age, clinicians look for signs of readiness to safely oral feed including, alert state, weight gain and stable respiration. However, age is not sufficient, preterm infants must develop in two aspects in order to attain a safe suck-swallow-breathe behavior for all three phase of the NS: (i) maturation (GA and PMA) and (ii) experience (14, 62, 64, 65).

During the oral phase, preterm infants have a lower frequency, volume, and negative pressure during NS compared to their term counterparts (41, 66). The preterm infant must experience oral feeding to learn and create the neuronal patterns required since maturation alone is not adequate. With sufficient maturation, preterm infants given training and oral feeding experiences as early as 32 weeks PMA, will progress to more mature sucking stages of 3–5 around 34 weeks PMA, less experience will delay this progress (51, 64).

The preterm infant must also coordinate the pharyngeal and esophageal phases. The timing of pharyngeal peak pressure and the relaxation of the upper esophageal sphincter (UES) must evolve through both maturation and experience as well. Usually by the time a preterm infant is 34 weeks PMA, given enough experience, pharyngeal pressure is at its peak and the UES is able to fully and rapidly relax open. Younger preterm infants are at risk for dysphagia until this time because the pharyngeal pressure is lower and the UES is slower to relax open and does not open completely (67).

There is relatively little known about NNS in preterm infants in large part because it does not necessarily have an implication for NS performance until about 38 weeks PMA (44, 65, 68, 69). It is important, however, as a therapeutic action for the infant to help promote regulation of state (calm, sleepy, alert, fussy, crying, etc.) and lessen distress (39, 44). Similarly to NS, NNS is present around 28 weeks PMA and the frequency, volume, and negative pressure increases as the preterm infant ages (69). The pauses between bursts during NNS become shorter and more regular (less variation) and the bursts have increased frequency of sucks and longer duration with increasing PMA (39).

## Assessments

Routinely for many decades, clinicians, often speech therapists or nurse feeding specialists, have used a gloved finger and inserted it into the infant's mouth to gauge their sucking ability by considering the strength, rhythmicity, frequency and duration of sucks and bursts (14). This method is used to assess if an infant is ready to orally feed; whether they are able to get nutrition or if there is another issue causing clinical symptoms (such as failure-to-thrive). It is also used by lactation consultants to evaluate latch and sucking to aid in successful breast-feeding. Yet, this is a subjective judgement highly dependent upon the clinician's experience, tactile sensitivity, and how long the infant sucks on their finger. Going one step further, the Infant-Driven Feeding Scale was developed in an attempt to quantify the subjective assessments of the rater (70).

The Neonatal Oral-Motor Assessment Scale (NOMAS), developed in the mid-1980s, is a common observational tool used to assess jaw and tongue movement with qualitative results of normal sucking, disorganized sucking, or dysfunctional sucking pattern (71–74). A dysfunctional pattern is believed to be a sign of neurological impairment (75, 76), however it is controversial (71, 74). One problem lies in the NOMAS relying purely on the training and experience of the rater performing the scale because it is only an observation of how the infant feeds. A second problem arises when the NOMAS is compared to a later neurologic assessment. There are many different neurologic assessments done at different ages, and depending on which of them the NOMAS is compared to (BSID most commonly, early motor repertoire, or MOS, CRIB, Dubowitz, and NNNS to name a few), this can change how well the NOMAS score predicts the outcome of the neurologic assessment. A third problem comes from the variable chronological and gestational ages of the infants and/or preterms used in each study; there is not a routine age when the NOMAS is performed, nor are there consistent longitudinal scores taken for each infant in each study. Clinicians are in need for objective measures of the sucking pattern of each neonate in order to tailor therapies to improve primary measures along with improving secondary outcomes, such as weight gain or increase in feeding volume.

## Quantitative Measures

One of the earliest known reports of quantifying neonatal sucking was published in 1865 by Herz, who measured negative intraoral pressure using a mercury manometer attached to a nipple (77). Other similar methods for quantifying early sucking can be found in the literature from the late 1800s through to the early 1900s [see (77) for review]. More recently, there has been an increasing number of technological solutions to advance the quantification of sucking activity (78).

**Figure 7:**
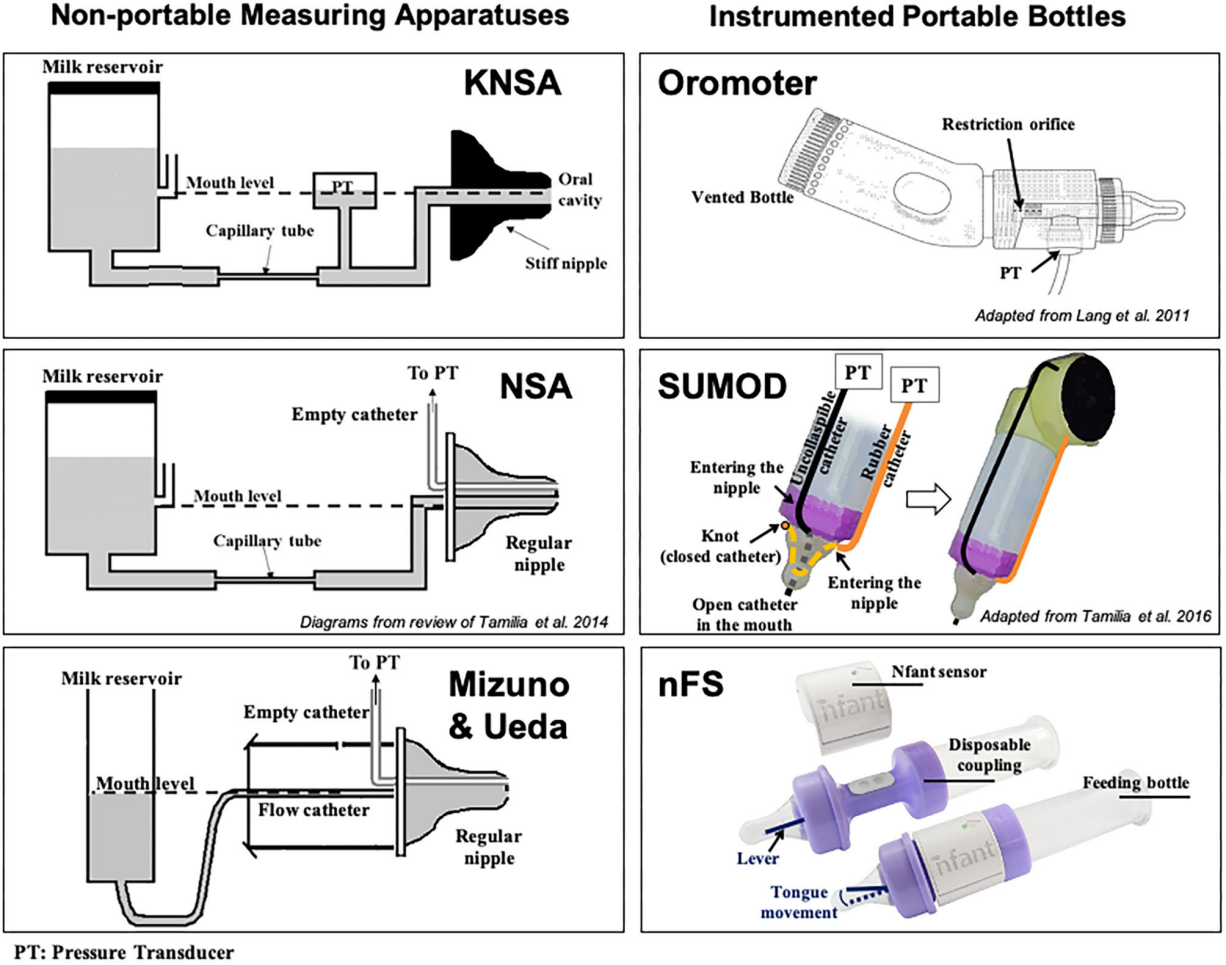
Technological solutions to assess neonatal sucking. Schematic diagrams of some technological solutions used to measure sucking during bottle feeding in infants. **(Left)** Non-portable measuring apparatuses [KNSA (77), NSA (79), and Mizuno-Ueda system (80)]: pressure transducers (PTs) are used to measure sucking pressures exerted on a nipple, which is not connected to a regular feeding bottle, but to a reservoir containing milk via a flow-regulating capillary tube or regular catheter. The milk reservoir is always kept at the mouth level to eliminate net hydrostatic pressure (unlike in regular bottle-feeding sessions). Regular nipples were used in NSA and Mizuno & Ueda's systems, while a stiff nipple was used in KNSA. **(Right)** Portable measuring solutions: feeding bottles instrumented with pressure transducers (PTs) to measure different sucking pressures [Orometer (81) and SuMOD (82): via air-filled catheters] or tongue movement (nFS; via a lever). These solutions were designed to be attached to regular bottles for easy use in clinical settings. Orometer measures the suction component of sucking; nFS measures tongue movement (related to the expression component); while SuMOD measures suction and expression components separately (enabling coordination assessment). nFS is a wireless solution, while Orometer and SuMOD require wired connection to an acquisition system.

Kron et al. published their seminal work for quantifying early sucking in 1963. Their interest in neonatal sucking was motivated by the desire to better understand the role of infant sucking in psychological development—specifically, personality formation (77). The researchers used a specially constructed nipple fused at the tip with rubber tubing that required active sucking (negative intraoral pressure) to release the flow of liquid. Continuous graphic output of the pressures within the mouth of the infant were recorded from a pressure transducer fixed between the flow device and the specialized nipple. Sucking variables included number of sucks per minute, volume consumed per minute and average pressure per suck, per minute. Based on results, the authors concluded that term infants altered their rate of sucking to increase the amount of liquid consumed on the second and third days of life. Further analyses suggested that infant sucking adaptations were a result of both maturation and a learning effect from the reinforcement of sucking at the breast or bottle. The instrumentation they used became known as the Kron Nutritive Sucking Apparatus (KNSA) (Figure 7).

Medoff-Cooper and colleagues used customized software to expand the sucking parameters that could be derived from the KNSA, in a 5 minute sucking session. These included number of sucks per session, sucking duration (interval from first to last suck in session), number of bursts in session (2-s pause defined separation of 2 bursts), mean burst duration, within-burst suck frequency, and mean maximum sucking pressure, among others (83). Following analyses, the authors concluded that for preterm infants, different aspects of sucking matured at different maturational ages. In a subsequent study by Bromiker et al. (84), suck rate, suck-to-burst ratio and time between bursts were used to investigate the influence of preterm infant feeding practice on feeding development at term. The authors found that earlier introduction of oral feeding resulted in greater feeding organization at term age; again highlighting the influence of learning and practice on the development of sucking skills.

Medoff-Cooper et al. went on to use the Nutritive Sucking Apparatus (NSA) (Figure 7), a modification of the KNSA, to investigate the relationship between preterm infant early sucking patterns and neurodevelopmental outcomes at 1 year corrected age (79). For this study, the researchers included a suck maturity index (SMI) to correlate sucking and developmental outcomes. The SMI was a composite score that included number of sucks, mean sucks per burst, and mean maximum pressure across all bursts. The authors found that sucking performance at 40 weeks PMA was significantly correlated to a standardized test of development at 1 year. They concluded that standardized assessment of neonatal sucking could serve as a means of early screening for the risk of developmental delay.

Ongoing modifications of the NSA have been published under the device name Medoff-Cooper Nutritive Sucking Apparatus (M-CNSA) (85). M-CNSA was used to investigate the effectiveness of a multi-sensory intervention on sucking organization of premature infants. The intervention group demonstrated significantly increased number of sucks, sucks per burst and maturity index by Day 7, as compared to premature infants receiving standard of care. The current evolution of the M-CNSA is now marketed under the device name Neonur (86), which houses a unit between a standard bottle and nipple that includes a pressure sensor, a signal processor and an on board flash memory drive. Following collection of sucking performance data, the memory drive data are downloaded to a PC and the data is processed via MATLAB^Ⓡ^ (MathWorks, Natick, Massachusetts). Sucking parameters recorded include those previously detailed in Bromiker et al. (84).

To better understand the relationship between sucking, swallowing and breathing, Mizuno and Ueda modified bottle nipples, routinely used in the nursery, to measure negative intraoral pressure (80). A silicone tube was inserted inside the nipple and connected to a microsemiconductor pressure transducer (Figure 7). Level of milk flow was dependent on the strength of the suction and expression component of the suck. They calculated sucking pressure, frequency, and duration as well as sucking efficiency. At the same time, they measured coordination of swallowing and respiration by recording pharyngeal pressure via an open silicone catheter placed transnasally at the oropharynx and connected to a transducer. Similar to Bromiker et al., they found that for healthy preterm infants, sucking pressure, frequency and duration matured with age. They also reported that the coordination of breathing and swallowing also matures with age.

In 2011, Lang et al. published work using the Orometer [(81); Figure 7]. The device they developed included an analytical system for analysis of suck data, the Suck Editor. They demonstrated the feasibility of their approach with a cohort of healthy term infants, confirming the work of others that specific aspects of sucking change with age. The authors concluded that quantitative measures of oral-motor function might serve as a proxy for neurodevelopment. In subsequent investigations using the Orometer and accompanying software, factor analysis of the more than 40 metrics collected by the system, identified seven factors that that best represent feeding skills as measured by the device: suck vigor, endurance, resting, irregularity, frequency, variability, and bursting (87). However, it is not clear the degree to which these specific measures might be sensitive to neurodevelopment.

Tamilia et al. advanced the field significantly by developing quantitative measures of sucking behavior that included indices reflective of motor coordination and control, which are particularly sensitive to neurological issues and overall neurodevelopmental status (88). Chief among these was a measure of movement smoothness. Smoothness is considered a characteristic of coordinated movement (89), and measures of smoothness have been used to quantify motor learning, development, and recovery in the reaching movements of healthy individuals, persons diagnosed with Parkinson's disease, and individuals post-stroke among others (90, 91). Smoothness is derived from the speed profile of a movement (88). Uncoordinated, immature movement is characterized by intermittent acceleration and deceleration—or multiple submovements—on the way to a target, therefore, the more intermittent accelerations and decelerations, the more “unsmooth” the movement (91). As with other cyclic and oscillating movements, Tamilia et al. emphasized the importance of analyzing and reporting the coefficient of variability of sucking parameters, rather than just the mean, since variability serves as a correlate of the organization and maturation of a motor skill (82). They also introduced novel measures to quantify the coordination between suction and expression movements by using a dynamic system approach, which allows investigation into the emergence of coordination patterns during infancy, and they showed how these measures may help characterize the feeding behavior of infants at risk for later neurodevelopmental delays. To measure both suction and expression, Tamilia et al. developed and used a portable sucking monitoring device (SuMOD), which was designed to be easily integrated on to any regular feeding bottle (Figure 7). Along with the device, they developed an analytical automated system for the data analysis (82).

Capilouto et al. reported the use of sonomicrometry to measure the resultant compressive forces applied to the nipple during non-nutritive and nutritive sucking (92). Their work represented a shift from measurement of intraoral pressures to a focus on the role of the lingual musculature in driving safe and efficient feeding. The approach was grounded in animal models of tongue muscle disuse atrophy that documented multiple changes in rat tongue musculature between dam reared rat pups and intravenously fed (IV) rat pups from the same litter (93–96). Following sacrifice and excision of tongue muscle the IV fed group was found to have significantly fewer tongue muscle fibers, smaller fibers, and fewer motoneurons driving the muscle. The researchers concluded that same thing might be happening with infants non-orally fed for an extended period of time; such as preterm or sick term infants.

To test their aims, Capilouto et al. instrumented a standard pacifier and a flow through nipple with piezoelectric crystals strategically located to enable direct measurement of nipple deformation kinematics in response to forces of the tongue. After controlling for weight and PMA, they found significant differences in tongue force during NS and clinically significant differences in posterior tongue thickness between full term and preterm infants beginning to feed. Full term infants demonstrated greater tongue force and greater posterior tongue thickness as compared to healthy preterm infants (92, 97).

The use of sonomicrometry to examine sucking performance presented a number of challenges including the cumbersome nature of the computer equipment required to collect the data which required multiple people at bedside. The unsustainability of this approach took the team back into the lab to consider alternative ways to measure tongue movement on the nipple. The result was nfant^Ⓡ^ Feeding Solution (nFS; NFANT Labs, Marietta GA, USA) (Figure 7), a non-invasive device for quantifying neonatal and infant sucking performance cleared by the FDA for use in the NICU. nFS consists of a disposable nfant coupling that connects a standard bottle to a standard nipple. The coupling houses a cantilever mechanism for measuring tongue movement on the nipple. The nfant SSB Sensor connects to the coupling and wirelessly transmits real-time data on nipple movement to a tablet via the nfant Mobile App. nFS addresses a significant limitation of other devices as the real-time feedback of sucking performance allows the healthcare team to see the immediate impact of an intervention to improve feeding (98). Following a feeding, waveforms of NNS and NS nipple movement are transmitted to the HIPAA protected nfant Cloud Database and the signals converted via custom algorithms, to identify key features and measures that describe sucking performance.

Recently, Capilouto et al. compared objective metrics of nutritive sucking performance via nFS between preterm and full term infants at discharge (99). They found that suck frequency accounted for 28% of the variance in feeding-related length of stay (FRLOS) for preterm infants, while suck smoothness accounted for 34% of the variance in FRLOS for full term infants. The researchers concluded that suck frequency may be an important intervention target to consider for preterm infants having difficulty transitioning to full oral feeding. They concluded further, that suck smoothness might be a sensitive marker for identifying infants at risk for feeding difficulties. The utility of nFS for quantifying sucking performance pre- and post- intervention has also been demonstrated (100).

## Sucking Activity In Infants With Brain Injury

The location in the brain stem of the CPG that controls sucking and swallowing corroborates studies that have found brain stem injury results in feeding difficulties. Quattrocchi et al. found a strong association between infratentorial, specifically brain stem, lesions on MRI and a diagnosis of oral motor dysfunction in infants with a hypoxic-ischemic injury (101). Because sucking and swallowing is a highly organized process that requires several different areas of the brain, injuries in other areas result in feeding problems as well. Martinez-Biarge found an association between basal ganglia and thalamic (BGT) and mesencephalic injuries with feeding impairment. Infants with severe BGT and mesencephalic injuries had an 84% probability of having a feeding impairment and severe BGT injury with pontine involvement had a 91% probability of getting a gastrostomy or having a nasogastric tube for at least 6 months (102).

Some longer-term studies have found a relationship between feeding performance and better neurodevelopmental outcomes. Mizuno and Ueda found a significant correlation between improved NS patterns over two examinations performed 2 weeks apart and better neurodevelopmental outcome at 18 months of age (103). Medoff-Cooper et al. measured NS parameters at 34 and 40 weeks PMA and found a positive correlation between a more mature pattern and a better BSID-II score (both MDI and PDI subscales) at 12 months of age (79). These evaluations of feedings were over a short period, demonstrating not only the rapid ability of an infant to learn to feed which speaks to neuroplasticity, but it also implies there may be a very short window of opportunity to improve outcomes for infants.

**Figure 8:**
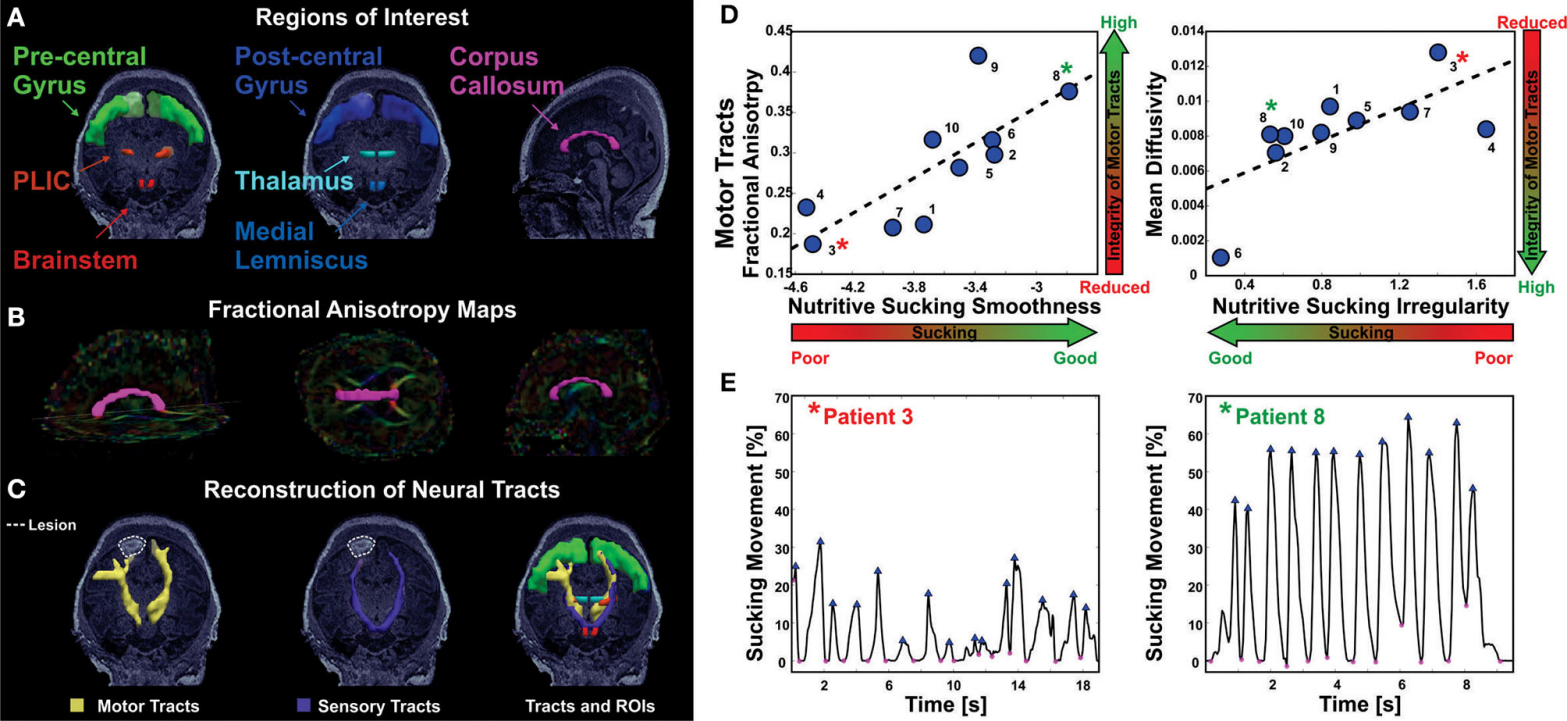
Correlation of abnormal NS patterns with integrity of sensorimotor fibers in infants with established in infants with established brain injury [from Tamilia et al. (31)]. **(A)** Anatomically defined regions of interest overlaid on the T1 MRI of a female 4 day old preterm infant. On the left, regions of interest for the motor tracts; in the middle, regions of interest for the sensory tracts; on the right, regions of interest corresponding to the corpus callosum. **(B)** Corpus callosum (magenta) overlaid on the fractional anisotropy color-maps. **(C)** Axial view of the motor (in yellow) and sensory (in purple) tracts reconstructed via probabilistic diffusion imaging tractography, along with the regions of interest used for their delineation. Neural tracts and regions of interest are overlaid on the patient's MRI that shows ischemic injury in the right frontal lobe. **(D)** The values of nutritive sucking smoothness and irregularity are predictive of the fractional anisotropy and mean diffusivity values, respectively, for the motor tracts. High smoothness in the nutritive sucking pattern, which is indicative of good sucking skills, is associated with high-fractional anisotropy, which is indicative of intact neural tracts. High irregularity in nutritive sucking, which is indicative of poor sucking skills, is associated with high-mean diffusivity, which is indicative of low integrity of neural tracts. **(E)** Two bursts of nutritive sucking from patients 3* and 8*. The left waveform demonstrates a poor NS behavior of patient 3* characterized by low smoothness and high irregularity (i.e., presence of multiple peaks); while the right waveform demonstrates a good NS behavior of patient 8* characterized by the smooth and regular nutritive sucking pattern.

Tamilia et al. demonstrated a correlation between microstructural abnormalities in the brain measured by MRI/DTI and sucking pattern variations. Specifically, motor tracts with poor integrity correlated with sucking patterns of lower smoothness and increased irregularity (31). This pilot study demonstrates the potential to identify brain injury through the analysis of nutritive sucking. Tamilia et al. used nFS to investigate the relationship between nutritive sucking and microstructural brain abnormalities (31). Using the accelerometer data captured via nFS, active feeding was analyzed using in-house software developed in MATLAB (82). Results indicated that specific sucking parameters were correlated with microstructural integrity of the sensorimotor tracts that control neonatal oral feeding (Figure 8). Specifically, low smoothness values as well as high sucking irregularity and low smoothness variability were associated with reduced microstructural integrity. Researchers concluded that quantitative assessment of sucking at the bedside could potentially result in earlier diagnosis of diffuse white matter brain injuries. Identifying brain abnormalities while in the NICU could serve to inform NICU care and take advantage of neural plasticity when the benefits would be greatest.

## Therapies

Infants that are unable to feed orally are deprived of the pleasurable oral sensation, instead they experience unpleasant sensations of a nasal-gastric tube, suctioning to prevent aspiration and/or tracheal intubation which makes them resistant, and even defensive, to oral feeding (58). Along with the motor restrictions this causes, the sensory inputs are detrimental to their oromotor development (14). It is vital to train infants to suck in order to feed orally and these are some examples of therapies applied: (i) *Kinesthetic:* a passive range of motion movement of the arms and/or legs during NNS. (ii) *Visual stimulation:* eye to eye contact during sucking. (iii) *Vestibular:* gentle horizontal rocking during sucking. (iv) *Auditory reinforcement*: an adapted pacifier is used to play soothing music (Pacifier Activated Lullaby, or PAL) or the mother's voice when the NNS reaches a threshold strength. The threshold can be increased as the infant gets stronger to further encourage development of a NNS (104). (v) *Sensorimotor stimulation*: A broad term that describes several techniques that can be used in various combinations to stimulate and/or reinforce an infant's suck. These techniques fall into one of three categories, oral/intraoral (O/IO), perioral (PO), or extraoral inputs and can vary between training times of 3–30 min, 2–4 times per day, for 10–14 days. O/IO includes gum and tongue stimuli ranging from a therapist's finger to a pulsating pacifier, PO consists of stroking or stimulating an infant's cheeks and/or lips, and extraoral entails tactile input to the head, neck, trunk, and/or limbs (54, 104). (vi) *Oral support*: The act of supporting the cheeks and chin during feeding. A novel example of this is using Kinesio Tape to apply a small force to a muscle by connecting the insertion and origin points to facilitate proper movement for sucking (105). (vii) *Swallowing program*: Placing a liquid bolus (either with a controlled flow nipple, a dropper or the like) on the tongue to stimulate a swallow response to encourage NS.

These therapies may be used in combination or sequentially to work toward the more advanced NS techniques. Fucile et al. found that oral (O) therapy for 15 minutes twice a day resulted in more mature sucking stages in both suction and expression components. However, tactile/kinesthetic (T/K) therapy of the same frequency and duration, did not improve sucking stages but did improve the swallow-respiration pattern by decreasing apnea-inducing pauses and increasing the safer, more adult-like, intra-expiration swallow pattern. Interestingly, combining O and T/K did not result in compounding effects probably because the frequency and duration of therapy did not change, only the type of therapy, therefore, the combination therapy preterm infants received half the amount of O and T/K therapies as their single-therapy counterparts (54). It appears that NNS training alone does not have a significant effect on primary outcomes of sucking measures, which may have consequences later in life such as speech and developmental delay (104). However, it does result in clinically significant secondary outcomes of reduced hospital stay, transition from tube to bottle, and improved feeding performance (increased milk volume) (106–109).

A commercially available “pulsating” pacifier used for training an infant to non-nutritively suck, the NTrainer, has been shown to be effective (110, 111). The logic behind the device is to stimulate the nerves involved in NNS and, thus, the CPG, in order to form a functional, effective NNS pathway using the principle “neurons that fire together, will wire together.” This therapy is effective in increasing the rates of NNS bursts, cycles, cycles per burst resulting in more daily oral feeds (110, 111). With the NTrainer, preterm infants showed an accelerated time to oral feed and time to discharge when it was used for 20 minutes up to four times per day until full oral feeding (112); however, in longer term follow up, NTrainer therapy did not result in improvement in cognition, language or motor skills (113). While NNS does accelerate the development of preterm infants while in the NICU, it does not appear to have any long term lasting effects on further brain development.

## Future Directions

Development of the field of brain neuroimaging in correlation with sucking patterns needs further confirmation and advancement. The quantitative study of sucking in many abnormal conditions could provide valuable insights to advance our knowledge as well as inform therapies. For instance, oligohydramnios or esophageal atresia that may prevent a fetus from swallowing *in utero*; what affect does this have on their NS and NNS ability as neonates. Are their effective therapies and why are they effective or not within the context of what we know about the development of sucking. This would also apply to the study of the sucking activity of neonates and infants with other conditions, such as Prader-Willi, Down syndrome or Spinal Muscular Atrophy (SMA), to name a few.

Bromiker et al. compared nutritive sucking parameters in Israeli and American preterm infants and found American infants had more mature NS patterns (more sucks, a higher rate of sucks, more sucks per burst, and a shorter interburst width) at 34 weeks PMA which the authors attributed to oral feedings being initiated on average almost 2 weeks sooner (84). This supports the notion that oral feeding training should begin very early, while the infant is still in the NICU. The concept of using different imaging modalities such as magnetoencephalography (MEG) or high-density electroencephalography (HD-EEG) to identify brain abnormalities that might be missed using more routine imaging such as cranial ultrasound and MRI should be explored. The passive techniques of HD-EEG and MEG are safe and effective and may show promise in this fragile population.

There are currently animal studies that are using positron emission tomography (PET) imaging to localize metabolically deficient areas of the brain caused by neuroinflammation, hypoxic-ischemic encephalopathy (HIE), and endotoxin exposure (114–116). The drawback with PET is the radioactive isotope exposure, however, these studies have had exposures 8–12x less than a CT scan. A potential expansion of the use of PET, which could complement MRI data, could be in neonates with brain injury. There is currently no data correlating PET and sucking activity in neonates leaving this a large potential area for exploration.

A recent study by Badran et al. shows promise in using non-invasive vagus nerve stimulation (VNS) that targets the auricular branch, transcutaneous auricular VNS, or taVNS. Fifty-seven percent of the infants in their study that had previously failed oral feeding therapies attained full oral feeds after an average of 16 days of taVNS treatment (117). The idea that VNS along with motor activity can stimulate neuroplasticity, improve motor function, facilitate neurogenesis and reorganization as well as restoring brain function in both human and animal studies, makes this a promising line of inquiry.

Another major area requiring development is in training a neonate to suck correctly, using *both* expression/compression and suction. Feeding training in NICUs currently focuses on secondary outcomes; weight gain, hospital discharge time, etc., with little regard for the primary ability to use both suction and expression/compression in a rhythmic fashion for a mature NS pattern. The next step beyond this would be exploring whether the proper NS pattern aids in repairing the brain injury, or at least re-wire the circuitry around the damage. Neuroplasticity in infants is also still being elucidated, perhaps this advancement could lend insight there as well. Inconsistent results for developmental scales and the like may also improve with a focus on primary measures of NS.

## Conclusion

The idea of a brain injury affecting the oral feeding of an infant has been around for decades. The flip side of that idea, the notion that we can identify a brain injury through analysis of how an infant sucks, could be instrumental in the identification of neonates in need of therapies and habilitation. Being able to do this very early in life, before any conventional scales or testing can be performed and even at the bedside while the neonate is still in the NICU, could take full advantage of neuroplasticity in early infancy and potentially guide clinicians in the repair of brain injury.

The window for training an infant to suck with a mature NS pattern is likely short, a few weeks, most of which could be done while still in the NICU. This concept of early oral feeding training could likely greatly diminish or even eliminate the need for ongoing therapy and compliance after discharge home. Only time will tell if a mature NS pattern will lead to better long term neurocognitive, speech, and developmental outcomes for infants with brain injury; however, having an infant that can orally feed efficiently would be a great step forward to ease the stress on families taking their infant home.

## Author Contributions

SS contributed writing content and creating figures. GC, DR, and YJ contributed writing and editing content. ET and CP contributed writing, editing content, and creating figures. All authors contributed to the article and approved the submitted version.

## Conflict of Interest

GC is employed by the company NFANT Labs, LLC. The remaining authors declare that the research was conducted in the absence of any commercial or financial relationships that could be construed as a potential conflict of interest.
